# Cancer incidence in Yemen from 1997 to 2011: a report from the Aden cancer registry

**DOI:** 10.1186/s12885-018-4411-9

**Published:** 2018-05-08

**Authors:** Amen Ahmed Bawazir

**Affiliations:** 1 College of Medicine and Health Sciences, University of Aden, Khormaksar, Aden Yemen; 2 Community and Environmental Health Department, College of Public Health and Health Informatics. KSAU-HS, Riyadh, Saudi Arabia

**Keywords:** Cancer, Registry, Incidence, Aden, Yemen

## Abstract

**Background:**

This study aims to report on the trend and incidence of cancers in Yemen (Aden) using data from Aden Cancer Registry (ACR), as a population-based cancer registry in Yemen over a period of 15 years (1997–2011). Such comprehensive, valid and detailed information on cancer trend is badly needed for planning a cancer control program in the country.

**Methods:**

All cancer cases were abstracted from patients’ medical records – based on clinical, histopathology, and radiological diagnosis. Data were coded using the International Classification of Diseases for Oncology (ICD-O) and the International Classification of Childhood Cancer (ICCC-3) to code childhood tumors. The CanReg4 program was used to analyze the data for 15 years study period.

**Results:**

A total of 6974 cases were included in this study, 47% were males and 53% females. The overall annual incidence rate was 21.6/100,000 populations; however, the incidence in males was little lower than in females (20.0 and 22.9 per 100,000 populations, respectively). The top five cancers among males were leukaemia (10.5%), nonhodgkin lymphoma [(NHL), 10.1%], colon (7.5%), Hodgkin diseases [(HD), 6.1%] and stomach cancer (5.1%). For females, breast cancer was the top (30.0%), followed by leukaemia (7.6%), NHL (6.6%), colonic (4.9%) and ovarian cancer (4.5%).

**Conclusion:**

Our findings reveal that, there is urgent need to commence the early screening of breast cancer due to its high frequency among Yemeni women. The government should give more support for cancer registries in the country to sustain its vital contribution to cancer care.

## Background

Cancer is a major public health problem worldwide, and a systematic analysis for the Global Burden of Disease Study (GBD) reported that in 2015 there were 17.5 million cancer cases worldwide and 8.7 million deaths. Moreover, there was an increase by 33% in the number of cases between 2005 and 2015 [1] . In 2012, the World Health Organization (WHO) reported that the incidence of cancer in developing countries is increasing, approaching 70% of cancer deaths worldwide [2]. Besides, it is estimated that the percentage incidence of cancer will increase by 2030 compared with 2008, and that this increase will be higher in low and lower-middle income countries (82%, 70%, respectively), compared to upper-middle (58%) and high-income (40%) countries [3].

The Republic of Yemen is a large country with various climatic, topographic, and environmental conditions. Its provinces are characterized by different social and genetic patterns. Cancer registry in Yemen is still a major challenge in the absence of national cancer surveillance, as the country lacks a National Cancer Registry Center (NCRC). According to the World Bank classification, Yemen is classed as among the lower-middle-income economies [4]. Although data from Western Countries are regularly included in the International Association of Cancer Registries (IARC) publications on cancer incidence, the same is not true about Low and Middle Income Countries (LMIC) where the incidence is often estimated, due to of the lack of high quality data from the existing Population Based Cancer Registries (PBCR) [5–7].

There are four cancer registries in the Yemen: the Aden Cancer Registry (ACR), the cancer registry at the National Oncology Center, Hadhramout Cancer Registry, and the more recent Taiz Cancer Registry; however the last three were being run as hospital registries. Nevertheless, not enough national cancer-specific statistics are available and information on cancer patterns is still very limited. The PBCR in LMIC, including Yemen, often struggle with inadequate health services, transient populations, lack of funding, shortage of trained personnel, incomplete or inaccurate data, and the difficulty in establishing a reliable and reasonable cancer registry in the nation [8–10]. As a result, registries, such as those for cancer are not placed high among their priorities for action.

The ACR, supervised by the Aden Cancer Centre at the Faculty of Medicine and Health Sciences at Aden University, established in 1997, is the first PBCR and a pioneer registry in Yemen. The registry was included in the International Association of Cancer Registries in 1998. The registry plays a leading role in analysing the cancer data and reporting it to the local and national governmental administrations, enabling them to develop effective and relevant future health plans. It also demonstrated the potential to collect informative data on cancer incidence in a low income country at a very low cost. Data from the registry were used to compile five-year reports on cancer incidence published in 2003 (period 1997–2001) [11], and in 2009 (period 2002–2006) [12]. These data was used to implement many cancer preventive and curative activities in the country. Moreover, other published reports were collected from other regions of Yemen that deal with specific types of cancer, such as breast, colorectal, childhood cancers, and others, which are mostly based on hospital registries [13–18].

Yemen, being a Middle Eastern country with many cultural characteristics in common, has experienced some economic, social, and cultural changes resulting in significant modification to lifestyle. The objective of the current study was to investigate the incidence of cancer as reported in the ACR for the 15-year period of 1997 to 2011. The study also identified the top ten cancers in the ACR, which were compared with trends in other selected countries, mainly in the Middle East. Such data could be used to extrapolate the cancer incidence in other regions of Yemen.

## Methods

### Site of the registry

**Fig. 1:**
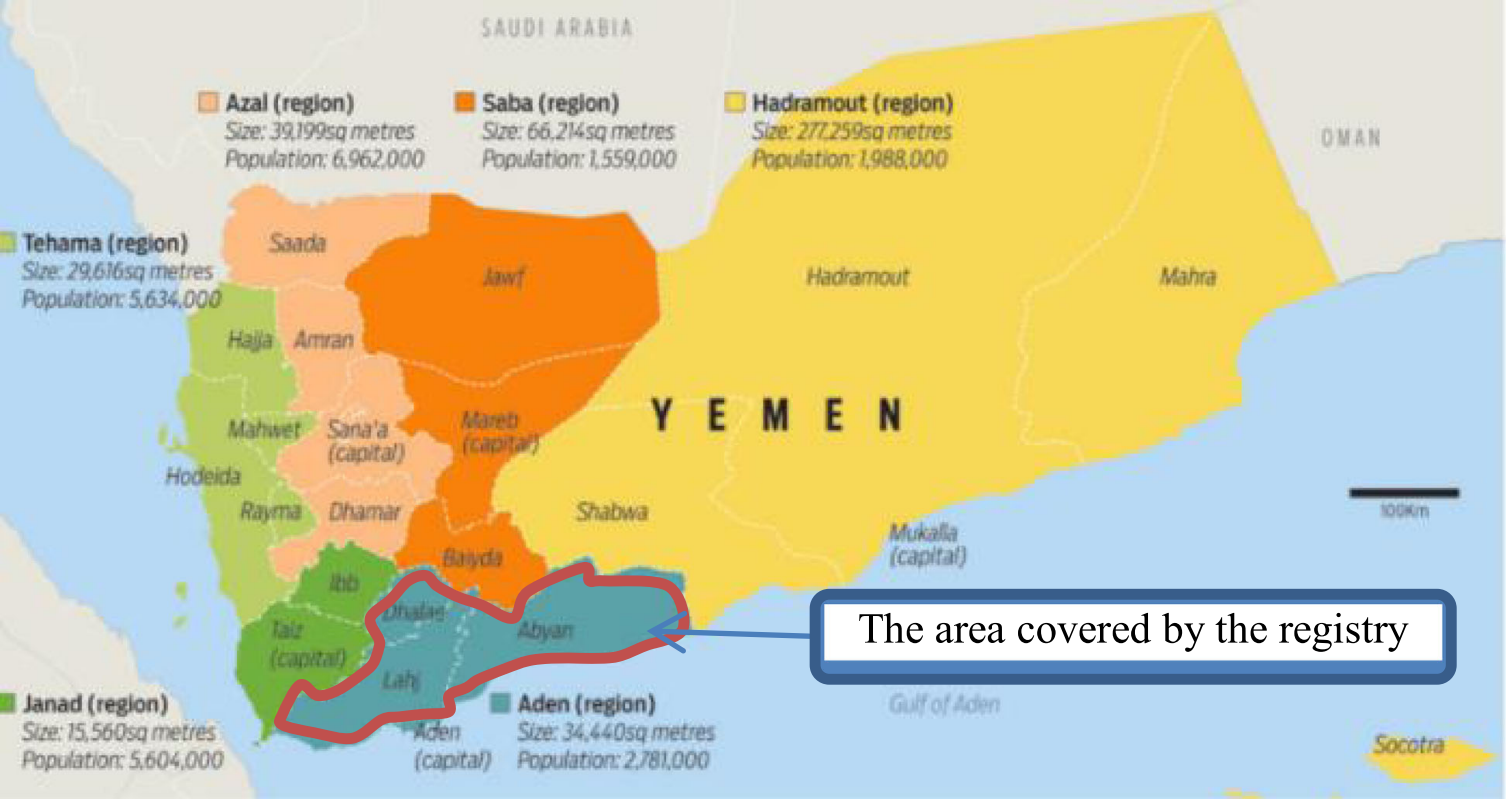
Map of the covered area of the registry (Aden, Lahej, Abyan, and Al-Dhale Provinces). Source: Ministry of Planning and International Cooperation, Yemen)

**Fig. 2:**
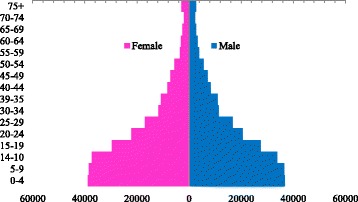
Estimated Midpoint (1997–2011) Population pyramid, Aden Cancer Registry Covering areas, Yemen. 1997–2011

The Aden Cancer Registry in Aden City is a PBCR, covering four administrative areas (provinces): Aden, Lahej, Abyan, and Al Dhale which cover a population of around 2,644,383 (Fig. 1.Population Pyramid) [12, 19], over a distance of 43,049 km^2^ (Fig. 2 Map of the coverage area). The data collected over a 15-year period (1st January 1997 to 31st of December 2011) for new cancer cases were used in this study.

Comprehensive cancer registration was achieved through data obtained from a combination of sources, such as outpatient clinic records, medical wards of the main seven hospitals (four in Aden and the rest from each referral hospital in the three provinces), haematology and histopathology laboratories, radiological/imaging diagnosis, clinical services, registry of the local cancer societies in each of the administrative areas, and from the registry of the cancer committee at the Ministry of Public Health for abroad referral. Relevant information on cancer cases was abstracted into predesigned registration forms and periodically submitted to the Cancer Registry office. Data entry, checks for completeness and accuracy were done using the CanReg-4 software from the International Agency for Research on Cancer (IACR), which was designed to input, store, check for duplicate registrations, validate and ensure consistency of the coded data, and then analyse the PBCR data [6]. The ACR adopted the International Classification of Diseases for Oncology, 3rd Edition (ICDO-3) for the classification of primary sites and morphology during the study period [20]. Although local laboratories were not able to identify the detailed subgroups of childhood tumours, such as leukaemia, the International Classification of Childhood Cancer (ICCC-3) code childhood tumours according to the availability of detailed findings, and mostly base this on tumour morphology [21]. Essential data were checked for duplication (by name, age, sex, and nationality), diagnosis, place of residence, and topography. Incomplete data were re-retrieved from the corresponding sources; however some cases were excluded from the study for incompleteness. Data in this study were received anonymous using a special code; hence there was no need for informed consent or ethical approval. The Cancer Research Center in Aden City as an academic unit in the College of Medicine, University of Aden acted as a formal ethics committee in in approving this study (CRD-13/#05). Mortality data were not made available to the cancer registry as registration of deaths seems to be both incomplete and inadequate with almost cancer is uncommonly stated as a cause of death and thus death certificate only (DCO) cases are not found in this data.

The registry defined multiple primary cancers according to the 2004 IARC/IACR rules, and were then recorded and tabulated accordingly. The registry adhered to IACR/IARC guidelines with respect to the preservation of confidentiality in connection with, or during, the process of collection, storage, use, and transmission of identifiable data.

Estimation of the population based on sex was obtained annually from the Department of Statistics, at the Ministry of Planning and International Cooperation (MOPIC-Yemen) for the intercensal period [19]. The incidence rate was calculated based on mid-time of the total population denominators of the covered area (2,644,383) for the period 1997–2011. The World Standard Population was also used for direct standardization, to calculate age-standardized rates per 100,000 populations. The age category classification of the IARC was used, based on age groups of five-year intervals (0–4, 5–9, 10–14, 15–19, 20–24, 25–29, 30–34, 35–39, 40–44, 45–49, 50–54, 55–59, 60–64, 65–69, 70–74, ≥75).

## Results

A total of 7413 records were reported as cancer patients (1997–2011). However, only 6974 (94.1%) were included in this study due to their completeness. The female to male ratio was 1.1: 0.9, with a total of 3690 (52.9%) females and 3284 males (47.1%). The crude rate (CR) for all cancer sites and the age-standardised incidence rates (ASR) during the whole 15-year period was 20.3 and 38.2 per 100,000 in males while in females was 22.9 and 36.1 per 100,000, respectively.

**Fig. 3:**
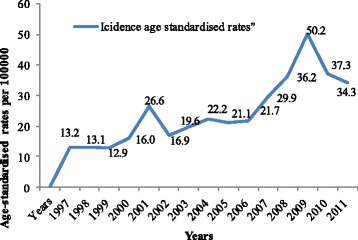
Age standardised incidence rates of cancer through the period of 15 years, ACR

**Fig. 4:**
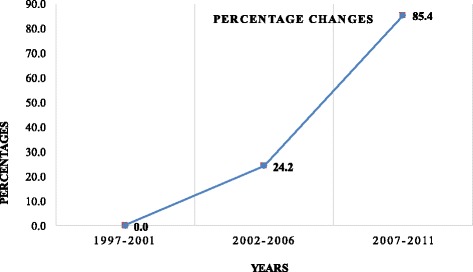
Percentage change of Age-standardised cancer incidence rate through 5 years intervals, ACR

The age-standardised incidence rates (ASR) of reported cases from the ACR for the 15-year period of 2005 to 2011 (Fig. 3) showed a steady increase of reported new cases. The calculated percentage change over 5-year period (1997–2011, 2002–2006, 2007–2011), using the first interval (1997–2001) as base-line point, showed a significant increase in the age-standardised incidence rates of reported cases; 24.2% more in the period 2002–2006, compared with 1997–2001, and with an 85.4% increase during the period 2007–2011 compared with 2002–2006 (Fig. 4).

**Fig. 5:**
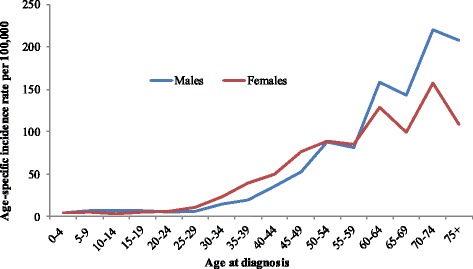
Age-standardised incidence rates rate of cancer among males and females in ACR

The peak of the age-specific incidence rate per 100,000 population was seen to be higher among females, predominantly in the young and middle age groups (30–59 years), but was then surpassed by higher incidences in males, particularly in those 60 years and older (Fig. 5).

### Five most common types of cancer (1997–2011)

**Fig. 6:**
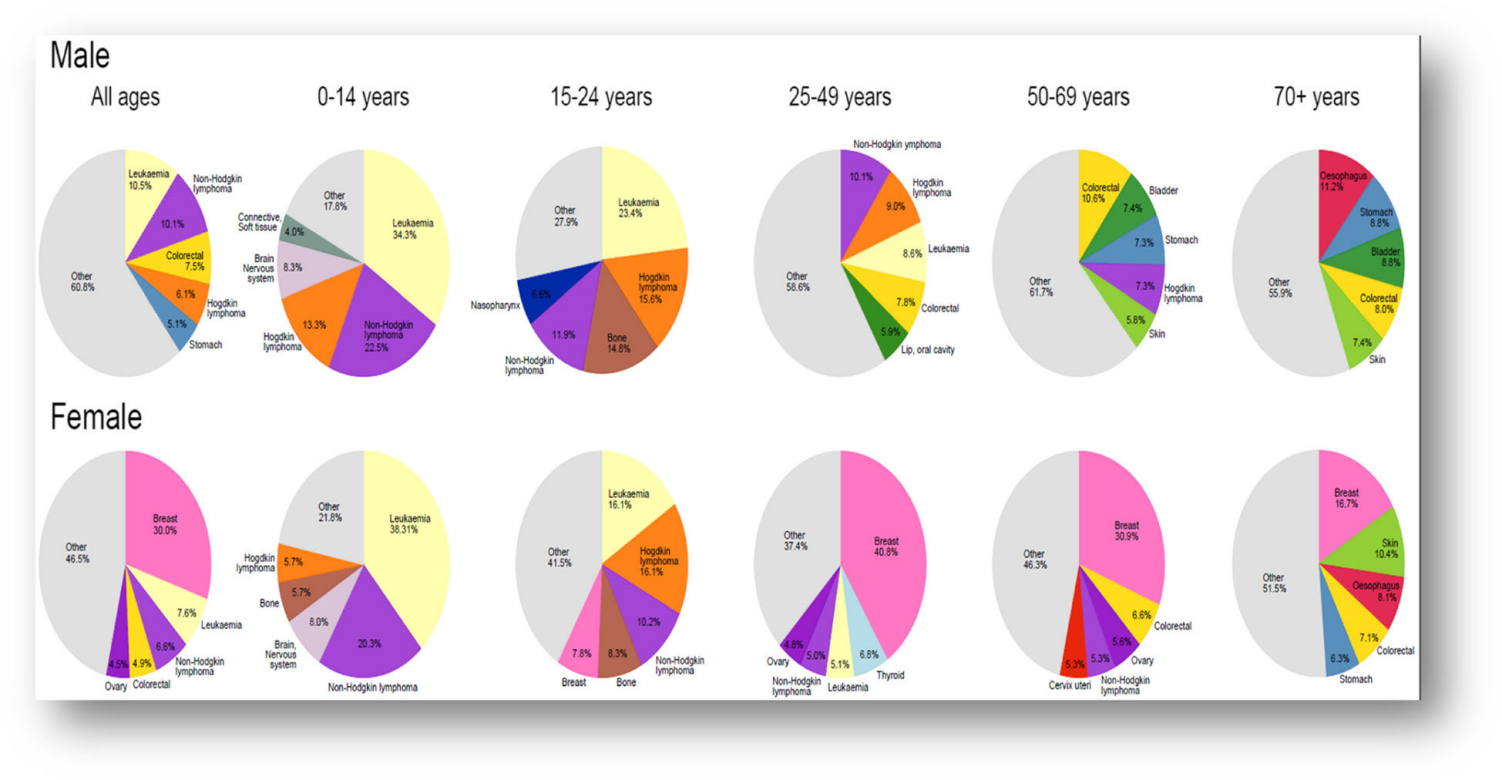
The most common five cancer types by gender and age category from the ACR (1997–2011)

For males, the five most common types of cancer were leukaemia (10.5%), non-Hodgkin lymphoma (NHL) (10.1%), colorectal (7.5%), Hodgkin diseases (6.1%), and stomach (5.1%). Whereas for females they were breast (30.0%), leukaemia (7.6%), NHL (6.6%), colorectal (4.9%), and ovarian (4.5%) (Fig. 6).

To understand the most common cancers in both the male and female study population, the cases were divided into five age-group cohorts. The following was the structure of the cohorts: children < 15 years old; adolescents and teenagers, 15–24 years; adults, 25–49 years; adults, 50 to 69 years; and elder adults, ≥ 70 years. Accordingly, each cohort showed some specifications in the most common type of cancer, with similarities or differences between males and females. For example, among males, leukaemia was the most common in children and teenagers < 25 years of age; NHL was the most common in the cohort belonging to the 25–49 year group; colorectal cancer was the most common type in the 50–69 year cohort; whereas oesophageal cancer was found to be the most common type of cancer among the elderly cohort aged ≥ 70 years.

Meanwhile in females it was found that leukaemia ranked as the most common type of cancer among the cohort of children < 15 years; Hodgkin disease was prominent among the cohort of adolescents and teenage females (15–25 years); breast cancer was the most common form of cancer in the other age-cohorts (25–49, 50–69, ≥ 70).

**Fig. 7:**
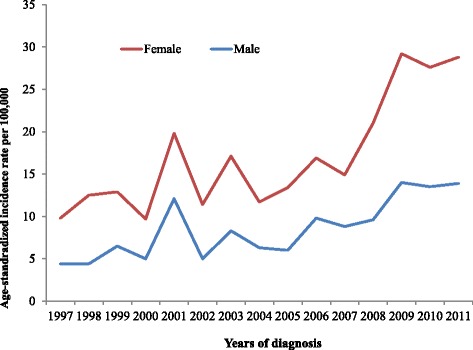
Age-standardised incidence rates rate of lymphomas in males and females over 15 years period in ACR

Comparing the proportion rates of similar types of cancers between males and females (as shown in Fig. 6), males showed higher rates of most cancers compared with females, except for breast cancer, which is sex specific and dominant in females. For example, the most common cancer in each age group was; leukaemia in children (34.3% (males) vs. 25% (females); Hodgkin disease in adolescents 15.6% (males) vs. 13.5% (females); NHL in the 25–49 year age group (10.1% (males) vs. 5% (females); Colorectal cancer in the 50–69 year age group (10.6% (males) vs. 6.6% (females); and finally oesophageal cancer in those ≥ 70 years (11.2% (males) vs. 8.1% (females). In the other hand, Fig. 7 showed the increased tendency of lymphomas over the period 1997–2011 with higher rates among females than males. Data in Fig. 7, showed that a steady increase in age-standardized incidence rate of non-Hodgkin lymphoma over the study period of this report (15 years) with some fluctuation in some years but with no significant differences.

## Discussion

The Aden Cancer Registry was established as a pioneer population-based cancer registry in Yemen staffed with qualified personnel trained by IARC in Lyon, France to run the cancer registry to the required standards. Data from this registry has shown a gradual increase in the overall annual incidence rates (crude and age-standardized), as well as an increase in the numbers of new cancer cases throughout the 15-year period of 1997 to 2009 (263 increased to 1004). Aden, in particular, was considered as a pioneer in providing public health services in Yemen; however recently, there is a growing trend for private hospitals, health centres, and clinics [22]. This growth in health services over the last 25 years has contributed to the increased reporting of cancer cases over the 15-year period in these areas, as well as in other parts of Yemen. From the very beginning, the Aden cancer registry was planned to be part of the national program of cancer control in Yemen. During all stages of its development it was gradually collaborate with many hospitals, cancer diagnostic laboratories, private cancer clinics and dispensaries in Aden city as well as in the administratively covered areas. In addition, over the 15-year study period Yemen has shown improvements in the implementation of different diagnostic methods for cancer, leading to an increase in the detection rate of cancer cases. Consequently, the quality of data registration was improved and also number of reported cases increased which reflected in the different increase through the calculation of the percentage change rates. Recent reports from countries in the region have shown a similar trend for increases in the reporting of cancer cases over a 10 to 15-year period [6, 23, 24]. Population growth and aging, combined with reduction in mortality from infectious diseases could explain the reasons for the worldwide increased cancer incidence [25]. It is worthwhile adding that changes in eating habits, tobacco use, and physical inactivity, or other factors that are already established in high income countries, such as environmental or socioeconomic development, were not adequately assessed in Yemen, and accordingly could not be of great contribution to this study.

In this study, no significant differences in cancer incidence were seen between males and females; however, the rate of cancer incidence in males was slightly lower than in females (20 vs. 22.9 per 100,000 world population). The age-standardized incidence rate of cancer for each gender in our study were very different to the incidence rates in some North African countries, such as Algeria, where the incidence rate reported by the cancer registry of Setif was 106.4/100,000 in men and 110.3/100,000 in women [26]. Over the 15-year period of 1997 to 2011, leukaemia was the most common cancer among males (10.5%), followed by NHL (10.1%), colorectal cancer (7.5%), Hodgkin disease (6.1%), and stomach cancer (5.1%); while among females the most common were breast cancer (29.9%), leukaemia (7.6%), NHL (6.6%), colorectal (4.9%), and ovarian cancer (4.5%). Summary statistics from the GLOBOCAN project on data in Yemen showed the five most frequent cancers among both sexes were breast (C50), leukaemia (C91–95), NHL (C82–85, C96), brain and nervous system (C70–72), and finally colorectal cancer (C18–21); however these rankings were inconsistent with those reported by the ACR, which were breast (C50), NHL (C82–85, C96), colorectal (C18–21), leukaemia (C91–95), and finally stomach cancer (C16). These differences were probably due to the data by the ACR being accumulative, and differences between GLOBOCAN and the ACR in the estimation time of the cancer cases [27]. However, GLOBOCAN estimated the length of time for each cancer case based on 2004 ACR data.

Excluding breast cancer in women, the three most common types of cancer in males overlap with those first reported in females (leukaemia, NHL, and colorectal cancer) in the study period. The rate of lymphomas in particular was found common in females than males which is not matching the rate found in some Central and south American countries where rates reported dominant among male patients [28]. Interestingly, the age specific pattern of Yemeni childhood cancer agree with the most common cancers reported in a previous study by Ba-Saddik (2013) [18], where leukaemia is the most common, followed by lymphoma and central nervous system tumours.

Studies from some registries in Middle Eastern countries, such as Jordan, Saudi Arabia and Egypt have patterns of cancer incidence that are different to our findings, except for breast cancer in females, which is the most common in many countries [23, 29, 30]. Surprisingly, the incidence of lung cancer is not highly reported in the ACR, which contradicts data reporting lung and colorectal cancer as the most common types of cancer in high income countries, and the Gulf of Bahrain [24, 27]. This could be due to the shorter life expectancy, and poor diagnosis of lung cancer in Yemenis, with more emphasis on tuberculosis, rather than cancer. However, the latest WHO report on the global tobacco epidemic, 2017 has reported an increased prevalence rate of tobacco smokers in Yemen, increasing by 18.7% [31]. Smoking in the country is usually associated with locally used leaves, named Khat (*Catha edulis*) [32–35], which could lead to an increase in the rate of lung cancer in the future.

**Fig. 8:**
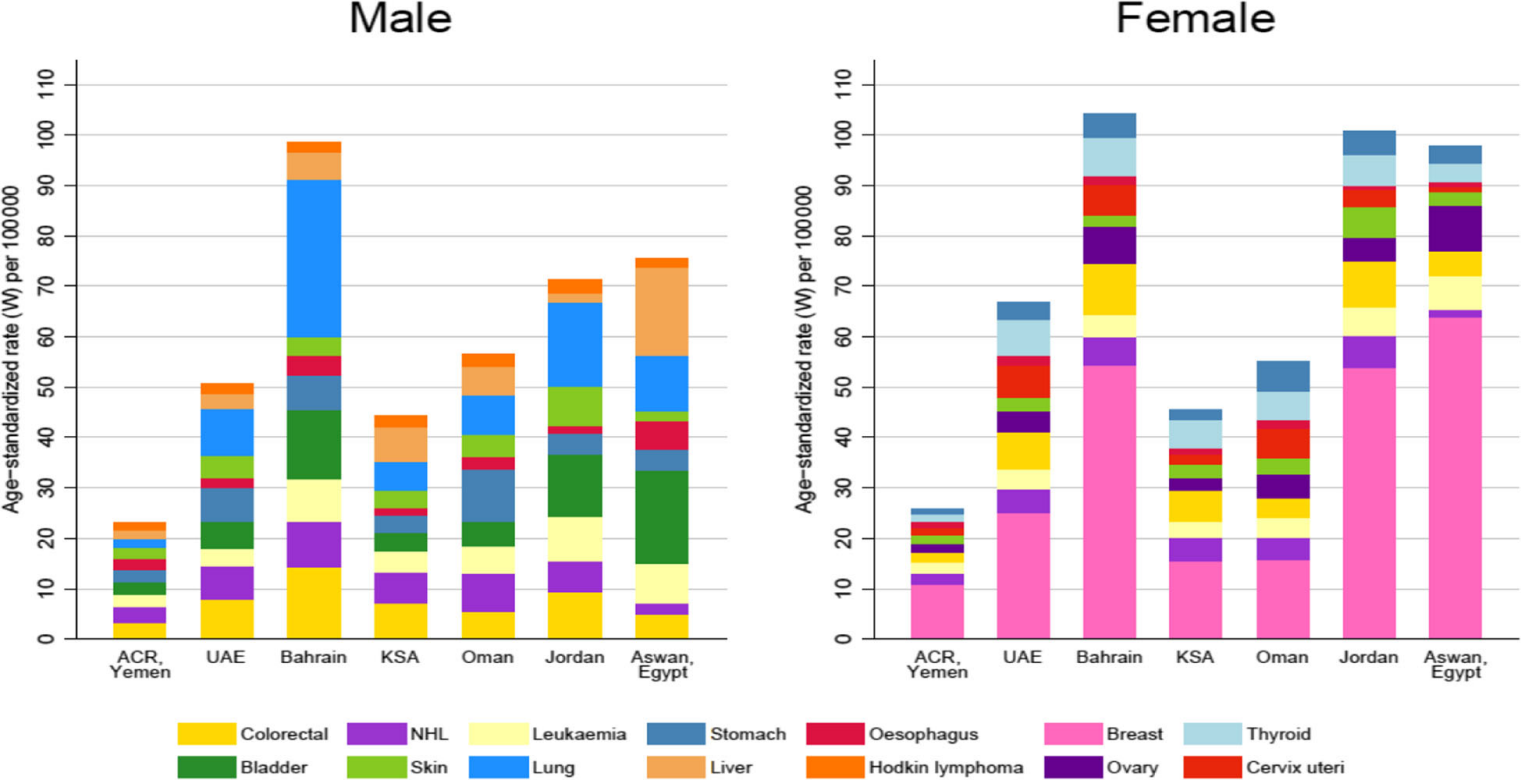
Comparison of ASR per 100,000 inhabitants of the top cancer in males and females for Aden Cancer Registry and selected countries

Figure 8, shows the ASR incidence rate of different types of cancers (per 100,000 populations) in both males and females from the ACR, as compared them to findings from some selected registries in the Middle East. The reported crude incidence (CR) rate from the ACR among males was 20.3, and in females 22.9 per 100,000 populations, which is disagrees with rates from other selected registries in the region. For example, the United Arab Emirates (UAE) has shown a CR of 38.3 among males and 45.2 in females per 100,000 population; Bahrain 96.3 among males and 106.3 in females; Saudi Arabia 40.3 in males, and 39.7 in females; Oman 56.1 in males and 51.3 in females; Jordan 66.2 for males and 70.0 in females; The Aswan registry in Egypt has reported a CR of 96.2 for males and 115.2 in females [23, 24, 36–38].

Recently, data from cancer registries in North Africa demonstrated acceptable quality with marked increase in regional coverage in these countries with compared cancer trend of one third and one half of what is observed in Europe [38].

According to this study, the overall ASR per 100,000 world population for males in the ACR (Yemen) was 38.2. This is very different to the results reported in many countries in the Middle East, including; AE (82.3), Bahrain (159), Saudi Arabia (59), Oman (92.1), Jordan (123.6), and Egypt (162). For females, the ASR per 100,000 world population in the ACR was (36.1) much lower compared with countries like UAE (95.1), Bahrain (154.6), Saudi Arabia (58), Oman (91), Jordan (122), and Egypt (122) [23, 24, 36–38].

In current study (ACR), the range of ASR among the ten most common cancer sites in males ranged from 1.5 for Hodgkin disease to 3.3 for colorectal cancer per 100,000 world population, whereas in females it was between 1.1 for stomach cancer and 11.0 for breast cancer. Although breast cancer (C50) was the most common cancer in females during the 15-year period (ACR data), the rate was the lowest compared with the selected countries in this study, such as UAE (25.1), Bahrain (54.4), Saudi Arabia (15.6), Oman (15.7), Jordan (40.9), and Egypt (57.8) [23, 24, 36–38]. Moreover, the ASR of breast cancer in the ACR was eleven times lower than what has been reported in the United Kingdom cancer registry (125/100000 vs. 11/100,000) [39]. Similarly, colorectal cancer in males from the ACR showed a much lower rate in comparison to the selected countries in this study; UAE (8), Bahrain (14.3), Saudi Arabia (7.2), Oman (5.5), Jordan (9.4), and Egypt (5) [23, 24, 36–38]. It is likely that the population in the selected countries with higher incidence rates of cancer undergo some form of screening and early detection for cancer, which is absent in Yemen. In addition, the healthcare services in areas covered by the ACR are not comparable with those in the selected countries where basic screening methods for breast cancer, prostate cancer, and colorectal cancer, for instance, are practiced on a daily basis, but this is not the case at the public primary healthcare level in Yemen [40].

We took note that the age trend for the occurrences of cancer at the ACR was different to others reported. For example, in Western registries, the peak age for diagnosing breast cancer is likely to be earlier among young women in the ACR, which is also similar to the age trend in the region [8, 24], compared with women in Western countries [41]. Interestingly, approximately 35% of women reported as having cancer were in the age group of 41–50 years. A study by El-Zaemey (2012) from the National Oncology Centre in Sana’a, Yemen, has demonstrated that approximately 71% of women diagnosed with breast cancer were 50 years old or younger [13]. Studies elsewhere have explained the role of reproductive factors, such as prolonged exposure to oestrogen, early menarche, late menopause, and others as contributing factors in the development of breast cancer [42–44]; however, these factors were not studied in our population.

According to the age groups (5-year intervals), three different age ranges were identified according to risk of developing cancer: children and adolescents had a very low incidence of cancer; 30 to 60 year olds showed an intermediate incidence, with highest peak being among females, which could be due to a peak in breast cancer incidence in this age-group. The final risk group belonged to the over 60 age group, with a predominance of cancer in males, rather than females.

This study has several limitations. Due to the descriptive nature of the study, it is only possible to speculate about the potential explanations for the observed cancer trends in Yemen. Individual-level environmental exposures and lifestyle-related factors were not recorded in the registry, nor the stage of cancer. Although incidence rates of new reported cases were dependent on the available diagnostic facilities, the quality of the cancer registry data did not provide information relating to mortality; this could hinder further the real incidence rate of cancer in the country. Similar situations could be seen clearly in many cancer registries in low resource countries, where attention usually focuses on the curative services rather than surveillance or prevention [5–7, 45].

Unfortunately, comprehensive data on cancer incidence covering the whole country is not yet available, because registries of the Ministry of Health in Yemen (2006) are only partially functioning, due to a lack of technical and financial support. However, the estimation of the country’s cancer profile would be possible if such data from the ACR were used to extrapolate Yemen’s cancer incidence. For example, the IARC’s GLOBOCAN estimates for cancer incidence in Yemen were derived from data available from the Aden Cancer Registry in 2004 [46]. A key provision in the resolution was to activate the existing comprehensive national cancer control programme, including the active role of cancer surveillance and integrate it into the country’s existing healthcare system. Setting up a cancer registry with limited resources has become a success story for the population-based Aden Cancer Registry and the attempt at providing good quality information on cancer patterns and trends has been promising. It was clearly reported that developing countries such as Yemen possess low accuracy of underlying cause of death in contrast to high accuracy in developed countries. In addition, many countries in Eastern Mediterranean Region and North Africa were lacked financial or technical resources to collect good quality, complete and timely data [38, 45, 47]. However, the use of death certificates and determining death rates caused by cancer may be an important step to strengthen the data quality reported from this registry.

## Conclusion

The findings of the ACR in Yemen (1997–2011) showed that the increases in reporting cancer incidence rates over the 15-year period were attributable to changes that occurred to the cancer registry’s functionality. It is also a result of the improved diagnostic methods used in different healthcare centres in the country. Data included in this study will provide the basis for evaluating priorities for cancer control in Yemen. It is also recommended that the different cancer registries in Yemen are activated to present the full picture of cancer in the country. Thus there is a need for redesigning, organizing and directing cancer registries in Yemen in the context of cancer control policies and programs.

## Abbreviations

ACR: Aden cancer registry
CR: Crude rate
DCO: Death certificate only
GBD: Global burden of disease study
HD: Hodgkin diseases
IARC: International association of cancer registries
ICCC-3: International classification of childhood cancer
ICD-O: International classification of diseases for oncology
LMIC: Low and middle income countries
NCRC: National cancer registry center
NHL: Nonhodgkin lymphoma
PBCR: Population based cancer registries
WHO: World Health Organization
